# Effect of ultrasound streaming on the disinfection of flattened root canals prepared by rotary and reciprocating systems

**DOI:** 10.1590/1678-7757-2016-0358

**Published:** 2017

**Authors:** Layla Reginna Silva Munhoz de Vasconcelos, Raquel Zanin Midena, Paloma Gagliardi Minotti, Thais Cristina Pereira, Marco Antonio Hungaro Duarte, Flaviana Bombarda de Andrade

**Affiliations:** 1 Universidade de São Paulo, Faculdade de Odontologia de Bauru, Departamento de Dentística, Endodontia e Materiais Odontológicos, Bauru, SP, Brasil

**Keywords:** Ultrasound, Enterococcus faecalis, Instrumentation, Irrigation

## Abstract

New technical and scientific developments have been advocated to promote the success of the endodontic treatment. In addition to rotary and reciprocating systems, irrigating solution agitation has been suggested and passive ultrasonic irrigation (PUI) is the most used. Objective: To evaluate, *in vitro,* the effect of ultrasound streaming (US) in the disinfection of flattened root canal systems prepared by the ProTaper, BioRaCe and Reciproc systems, utilizing the microbiological culture. Methodology: Extracted human mandibular incisors (n=84) were used. Suspensions of *Enterococcus faecalis* (ATCC 29212) were standardized and inserted along with the teeth immersed in brain-heart infusion (BHI) broth. The contamination was made following a protocol during 5 days. The teeth were randomly divided into six groups: G1, ProTaper Universal; G2, ProTaper Universal with US; G3, BioRaCe; G4, BioRaCe with US; G5, Reciproc; and G6, Reciproc with US. Irrigation was performed with saline solution. After biomechanical preparation, microbiological samples were performed with sterilized paper points, which were diluted and spread on BHI agar; after 48 h, the colony forming units (CFU/mL) were counted for each sample. Results: Groups using ultrasonic agitation presented a greater antibacterial effect than the other ones, even using saline solution as irrigant. The ProTaper Universal system showed the best antibacterial activity of the tested systems (median of 0 CFU/mL with and without surfactant or ultrasonic activation [PUI]). Even with PUI, Reciproc (median of 2.5 CFU/mL with PUI and 5 without it) could not reduce as many colonies as ProTaper Universal without US. The BioRaCe system had greater bacterial reduction when using US (median of 0 CFU/mL with PUI and 30 without it). Conclusions: US promoted greater reduction in the number of bacteria in the flattened root canals prepared with nickel-titanium mechanized systems. Regarding the instruments used, the ProTaper Universal system was the most effective in reducing the bacterial number.

## Introduction

Biomechanical preparation plays an important role in eliminating bacteria and reducing their population inside the root canal system. Teeth that have a complex anatomy can shelter, in the crevices and isthmus areas, remaining necrotic pulp tissue and bacterial biofilm, which can act as a potential source of persistent infections, resulting in the failure of endodontic treatment^23^. In these cases, Gram-positive microorganisms are the most frequent, and among these, *Enterococcus faecalis* ^5^ is the most commonly used. This bacterial species has the ability to endure many ecological conditions and it can adjust to lethal challenges such as high levels of alkalinity^23^, requiring few nutrients, adhering to dentine^21^ and penetrating deeply into the dentinal tubules^11^ ^,^ ^14^, which makes it a resistant pathogen^12^ and the microorganism of choice in antimicrobial studies in endodontics.

New technical and scientific developments have been advocated to promote the success of endodontic treatment. In addition to rotary and reciprocating systems, irrigating solution agitation has been suggested and passive ultrasonic irrigation (PUI) is the most used^29^. This kind of irrigation has shown better results in cleanliness and disinfection^7^. However, in PUI, physical action without chemical action of the irrigants has not been studied.

The nickel-titanium instrument (NiTi) ProTaper Universal (Dentsply Maillefer, Ballaigues, Switzerland) is made by machining. The instruments of the BioRaCe system (FKG, La Chaux-de-Fonds, Switzerland), also machined, were launched with electrochemical surface treatment, providing the removal of surface defects that can initiate a fracture when the instrument is subjected to a cyclic fatigue process^13^. The Reciproc system (VDW, Munich, Germany), which consists of a single NiTi instrument, has gained popularity in clinical practice due to its reciprocating movement. It was launched with the aim of reducing endodontic treatment time without altering its effectiveness^3^.

Thus, the aim of this study was to evaluate the effect of ultrasound streaming (US) in reducing microorganisms in the root canal system of flattened teeth prepared by the ProTaper Universal, BioRaCe and Reciproc systems, to assess the best clinical protocol to promote greater root canal system decontamination, since these teeth are more difficult to clean. The null hypothesis is that US does not favor greater disinfection in root canal systems of flattened teeth, as well as that the systems have the same effectiveness.

## Material and methods

### Specimen preparation

This study was approved by the Research and Ethics Committee of the local university (Number: 180/2011). Eighty-four extracted human mandibular incisors were used. The teeth had been extracted for pulpal or periodontal reasons. Radiographs in both directions were taken to select teeth with flattened but single canals. The selected teeth had a length of 18 to 22 mm and they were randomly distributed to all groups.

All teeth were scaled and stored in 1% sodium hypochlorite for 48 h to promote disinfection and dissolution of organic tissues. Conventional access cavities were prepared using round burs and Endo-Z burs (Dentsply Maillefer, Ballaigues, Switzerland). Canals were evaluated for apical patency with a size-10 K-file and instrumented to a size-20 K-file (Dentsply Maillefer, Ballaigues, Switzerland) 1 mm from the root apex and irrigated with 5 mL of saline solution. Then, specimens were submitted to three ultrasonic baths of 10 min each one with 1% sodium hypochlorite, 17% EDTA and saline to neutralize the anterior substances following the Marinho, et al.^16^ (2014) protocol. External surfaces of all roots were sealed with nail polish to allow bacterial penetration only by the crown access and the apical foramen, procedure confirmed in a pilot study.

After complete drying of the nail polish (24 h), specimens were individually placed in microtubes (Axygen, Union City, CA, USA) containing 1.5 mL of BHI broth (BD, Le Pont de Claix, Ròdano-Alpes – Isère, France) and autoclaved.

### Specimen contamination

The bacterial reference strain from the American Type Culture Collection (ATCC) number 29212, of *Enterococcus faecalis* was obtained. The colonial morphology evaluation and Gram stain were performed to confirm the purity of the strain at several times during the experiment.

The microorganisms were cultivated in BHI broth with successive subcultures to achieve exponential growth. Dilutions were made based on the absorbance value, obtained by turbidity measured in the spectrophotometer SF325NM (Bel Photonics do Brasil Ltda, Osasco, Brazil) until the right concentration was achieved.

The tracer microorganism contamination was made for a 5-day period at 37°C with aseptic and periodic culture media changes to maintain viability, following the Ma, et al.^15^ (2011) sequence of centrifugations and the Andrade, et al.^4^ (2015) protocol, and a Scanning Electronic Microscopic was used to confirm the bacterial colonization.

### Instrumentation procedures

The sterilized specimens were divided into six groups according to the instrumentation system used for root canal preparation, as follows:

G1: ProTaper U (Dentsply Maillefer, Ballaigues, Switzerland; n = 10):

Instrumentation with ProTaper U was made using the crown-down technique according to the manufacturer instructions until the F2 instrument was at working length (Sx, S1, S2, F1 and F2). For every instrument change, the irrigating solution was renewed.

G2: ProTaper U with ultrasonic agitation (n=10):

Instrumentation was performed in the same way as for G1; however, for every instrument change, the irrigating solution was activated for 1 min with a plain insert in a piezoelectric ultrasound.

G3: BioRaCe (FKG, La Chaux-de-Fonds, Switzerland; n = 10):

BioRaCe system was used with the crown-down technique and followed the manufacturer instructions until the BR3 instrument was at working length (BR0, BR1, BR2 and BR3). For every instrument change, the irrigating solution was renewed.

G4: BioRaCe with ultrasonic agitation (n=10):

Instrumentation was performed in the same way as for G3; however, for every instrument change, the irrigating solution was activated as it was for G2.

G5: Reciproc (VDW, Munich, Germany; n=10):

Instrumentation was performed with the reciprocating system using the crown-down technique and following the manufacturer instructions until the 25/.08 instrument was at working length. Before and after the instrumentation, the irrigating solution was renewed.

G6: Reciproc with ultrasonic agitation (n=10):

Instrumentation was performed in the same way as for G5. Before and after the use of the instrument, the irrigating solution was activated according to groups 2 and 4.

Two sterilized teeth *per* group were not submitted to contamination protocol and were considered negative control. As positive control, two teeth *per* group were submitted to contamination protocol but were not instrumented, proving the standardization of the initial contamination^4^.

The same operator performed all procedures. Every root canal was irrigated with a total of 10 mL of sterilized saline solution between each instrument with a NaviTip needle of 21 mm and 30 ga of diameter (Ultradent, South Jordan, USA) positioned at 3 mm short of the working length. In groups 2, 4 and 6, the irrigant was dispensed before ultrasonic agitation. For the ultrasonic agitation, an ultrasonic device activated by a piezoelectric ceramic pellet system at a frequency of 30,000 Hz (Jet Sonic, Gnatus, São Paulo, Brazil) by a plain insert (TU13, Trinit Periodontology, São Paulo, Brazil) for 1 min was used, in all ultrasonic groups; the procedure was conducted with vertical movements in the buccal-lingual and mesial-distal directions (30 seconds for each direction) in "endo mode" (50% potency). All experiments were performed under aseptic conditions in a laminar flow chamber to prevent airborne bacterial contamination.

### Sample collection

Teeth crowns were decontaminated by a swab soaked in 5.25% sodium hypochlorite for 30 seconds and neutralized with 5% sodium thiosulfate. Bacterial samples were collected by two absorbent #20 paper cones (Dentsply Maillefer, Ballaigues, Switzerland) from the root canal, taking 1 min for each cone, and then transferred to microtubes with 1 mL of BHI broth. Microtubes were agitated in a vortex for 10 seconds and 100 mL of the content of each tube was transferred to other microtubes, until it reached the 10^-4^ concentration. Aliquots of 100 mL of the dilutions were seeded in Petri dishes with BHI-agar broth. The dishes were stored in a bacteriological incubator for 48 h before the counting of the CFU/mL.

Data were collected, inserted in a spreadsheet and statistically analyzed using the SPSS 15.0 software. To compare the CFU/mL between the different instrumentation systems, the Kruskal-Wallis test was used for general and Dunn's test for individual comparison of the groups, with a significance level of 5%.

## Results

All instrumented teeth showed a bacterial reduction in the root canal when compared with the positive control (median of the control = 100 CFU/ mL). The groups using ultrasound agitation showed a significantly greater reduction of microorganisms when compared with the groups without ultrasound agitation, except the ProTaper group that showed the same results with and without the ultrasound agitation (median = 0 CFU/mL). BioRaCe groups presented a median of 0.0 CFU/mL with PUI, and a median of 30.0 CFU/mL without PUI. Reciproc groups had a median of 2.5 CFU/mL with PUI and a median of 5.0 CFU/mL without PUI.

Regarding the tested systems, ProTaper U showed a statistical significant difference when compared with BioRaCe and Reciproc groups without ultrasound, and also, when compared with the positive control (*p*<0.05). There was a statistical significant difference between BioRaCe instrumentation with and without ultrasonic use (*p*<0.05). When BioRaCe and Reciproc groups were compared with each other, there was not statistical significant difference (*p*<0.05). Also, there was not statistical significant difference between BioRaCe and Reciproc groups without ultrasound and the positive control (*p*<0.05). There was not statistical significant difference between the Reciproc instrumentation with and without ultrasonic agitation (Table 1).

**Table 1:** Median, minimum and maximum CFU/mL after the biomechanical preparation of each group, with and without ultrasound. Different lower-case letters indicate different statistical significances

Groups	Without Ultrasound	With Ultrasound
BioRaCe	30.0 (3.0-122.0)b,c	0.0 (0.0-3.0)^a^
ProTaper	0.0 (0.0-23.0)^a^	0.0 (0.0-3.0)^a^
Reciproc	5.0 (0.0-64.0)a,b,c	2.5 (0.0-27.0)a,b
Positive control	100.0 (100.0-100.0)^c^	100.0 (100.0-100.0)^c^
Negative control	0.0 (0.0-0.0)^a^	0.0 (0.0-0.0)^a^

a,b,c Different letters indicate statistical differences among the groups

**Figure 1:**
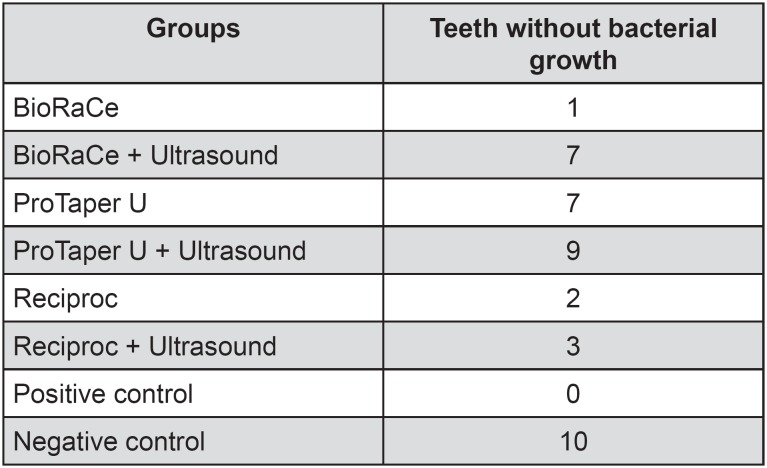
Number of teeth without bacterial growth for each group

In the group of BioRaCe system without ultrasonic agitation there was one specimen without bacterial growth, while when the ultrasound was used with the same system 7 specimens did not present bacterial growth. In ProTaper U group, 7 specimens did not present bacterial growth, but when the ultrasound was used this number raised to 9. The Reciproc system group presented 2 specimens without bacterial growth without ultrasound agitation and 3 specimens with no bacterial growth when the ultrasound was used. No specimens of negative control presented bacterial growth. Only 3 specimens of the BioRaCe system group without ultrasonic agitation presented more than 100 CFU/mL, as well as all the specimens from the positive control (Figure 1).

## Discussion

This study evaluated the effect of the ultrasound streaming in the disinfection of root canals. The null hypothesis tested was rejected since the ultrasound agitation favored a greater disinfection after the instrumentation with rotary system in flattened root canals and the systems had different antimicrobial effectiveness.

Similar to the Ma, et al.^15^ (2011) *in vitro* study, centrifugations were performed during the contamination of the teeth to fill the dentinal tubules with *E. faecalis.* However, in this study, more centrifugations were made, based on the Andrade, et al.^4^ (2015) protocol, as the entire tooth was used, not only a dentin block. Roots of all teeth were sealed with nail polish to ensure that the contamination penetrated only through the access cavities.

An efficient chemical and mechanical preparation is essential to decontamination of the root canal, eliminating bacteria and their byproducts, pulp and contaminated dentin^6^. Shaping and irrigation with antimicrobial solutions are able to reduce or eliminate the number of bacteria inside the root canals^26^ ^,^ ^27^. However, anatomical complexities can reduce the cleaning effects of the instrumentation and irrigation^2^. Studies have shown that approximately one-third of the canal walls are not touched by the instruments^19^ and that even the touched walls are not free of bacteria^17^. Shaping by hand or with NiTi rotary instruments has a limited effectiveness in flat canals, in which 30 to 40% of the root canal walls are not touched^19^. The NiTi rotary instruments promote a circular preparation, leaving the buccal and lingual extensions with debris^25^. Thus, mandibular human incisors were chosen for this study due to their flattened conformation.

In this study there were a great number of canals with negative cultures. The probable reason for this is that the sample collection was made only in the main root canal with absorbent paper points, so, bacteria may still remain inside dentine deep tubules.

PUI is more effective than conventional irrigation in cleaning the root canal system^25^ ^,^ ^29^. This kind of irrigation has the potential to remove dentin debris and organic tissue from areas inaccessible to instrumentation^29^. Rödig, et al.^24^ (2010) showed that PUI was more effective than irrigation with a syringe and a sonic system in the removal of debris in canal irregularities, with a complete removal of debris in 92.5% of samples. When pulp remnants and debris accumulation are present, bacteria come to harbor these materials. Because of that, it is rational to suppose that elimination of debris can collaborate to microorganism elimination. In this study, approximately 99% of the bacteria were eliminated in the groups with ultrasonic agitation.

Most of the studies that have investigated the antimicrobial effectiveness of PUI have used an antimicrobial solution as an irrigant^2^ ^,^ ^24^. In our study, saline was used to observe only the physical effect of PUI and the ability of different mechanical instruments to promote disinfection. Even without an antimicrobial solution, the instrumentation was able to reduce the number of bacteria in the root canal. When the preparation was associated with the ultrasound, results showed elimination of almost all bacteria, even with the innocuous irrigant. Carver, et al.^8^ (2007) showed that the addition of ultrasound promoted a sevenfold reduction in CFU/mL. After the root canal preparation, PUI allows the insert to freely swing inside the canal, thus causing cavitation and the physical disruption of the bacterial biofilm^1^ ^,^ ^20^. In this study, the PUI was used after each instrument due to the fact that instrumentation produces debris and smear layer, which, clinically, can be a protocol.

The rotary systems ProTaper U and BioRaCe and the reciprocating system Reciproc were chosen due to their different manufacturing methods, sections and protocols of use^3^ ^,^ ^9^, which can influence the ability to decontaminate the root canal.

The ProTaper U system showed the smaller median of CFU/mL when compared with the BioRaCe and Reciproc systems. The ProTaper U, when contrasted to other NiTi rotary systems, promoted a more aggressive dentin cut, which led to a greater bacterial reduction^10^ in agreement to other studies^9^ ^,^ ^18^. Besides promoting greater dentin removal, this system has more instruments than the others tested, which leads to a greater amount of irrigating solution and ultrasound used during the preparation.

All the systems have the same 0.25 mm apical diameter, but their tapers vary, which can influence results. In the ProTaper U system, the F2 instrument has a taper of 0.8, the same as the Reciproc system's 25/.08 file. In the BioRaCe system, however, the BR3 file has a taper of 0.4. Thus, the BioRaCe system wore away a smaller dentin area, resulting in less decontamination. In addition, when Reciproc was compared with ProTaper U, the latter showed a greater removal of dentin^7^, which can explain the results. On the other hand, the number of files used in each group is different: 5 in the ProTaper U group, 4 in the BioRaCe group and 1 in the Reciproc group, and by the fact that the ultrasound was used after each file, this leads to different overall time regarding its use and it can explain the given results.

## Conclusion

US promoted greater reduction in the number of bacteria in the flattened root canals prepared with nickel-titanium mechanized systems. Regarding the instruments used, the ProTaper Universal system was the most effective in reducing the bacterial number.
